# What an emergency physician needs to know about acute care of cardiac arrhythmias

**DOI:** 10.4103/0974-2700.62109

**Published:** 2010

**Authors:** Christoph Stellbrink

**Affiliations:** Department of Cardiology and Intensive Care Medicine, Bielefeld Medical Center, Germany

**Keywords:** Arrhythmias, emergency treatment, antiarrhythmic drugs

## Abstract

The treat of cardiac arrhythmias has been studied extensively in the last decades. There has been a major shift in antiarrhythmia treatment from drugs to interventional electrophysiological procedures and implantable devices. Published data indicate that for long-term treatment of arrhythmias, non-pharmacological treatment is more effective than drugs in many patients. Similarly, the overhelming success of radiofrequency catheter ablation of supraventricular tachycardias has almost eliminated the need for chronic drug treatment. Today, catheter ablation plays an increasingly important role in the prevention of atrial fibrillation recurrences. However, in the emergency room or in the intensive care unit, drug treatment remains the gold standard for the treatment of cardiac arrhythmias. Arrhythmias are very common in emergency medicine, occurring in 12% to 20% of all patients in an intensive care unit and there is great need for good diagnostic and therapeutic algorithms to aid the emergency physician dealing with patients suffering from arrhythmias

The treatment of cardiac arrhythmias has been one of the most active fields of scientific development within cardiology during the last decades. Since the publication of the results of‘ the Cardiac Arrhythmia Suppression Trial (CAST),[1] there has been a major shift in antiarrhythmia treatment from drugs to interventional electrophysiological procedures and implantable devices. The fundamental paradigm shift in thinking after CAST was that drugs which are meant to suppress arrhythmias may actually increase mortality in the long term because they are inherently proarrhythmic. Since CAST, a large volume of data concerning chronic antiarrhythmia treatment has been published. These data indicate that for long-term treatment of arrhythmias, non-pharmacological treatment is more effective than drugs in many patients. For example, data from the trials comparing standard drug treatment to the implantable cardioverter- defibrillator (ICD) in high-risk groups such as those with chronic heart failure[2] or reduced left ventricular function in the chronic phase after a myocardial infarction,[3] indicate a reduction in mortality in those patients implanted with an ICD both for primary and secondary prevention[4] of sudden cardiac death. However, there are some exceptions, namely patients early after a myocardial infarction[5] or patients with reduced left ventricular pump function undergoing coronary artery bypass grafting.[6]

Similarly, the overwhelming success of radiofrequency catheter ablation of supraventricular tachycardias has almost eliminated the need for chronic drug treatment in patients with atrioventricular nodal re-entrant tachycardias, accessory pathways, or ‘common-type’ atrial flutter. In atrial fibrillation (AF), the most common supraventricular arrhythmia, antiarrhythmic drug treatment is still a viable option for many patients. But catheter ablation plays an increasingly important role also in the prevention of AF recurrences, especially in young, highly symptomatic patients with the paroxysmal form of AF.[7,8] At the other end of the spectrum, elderly patients with minor symptoms may benefit more from adequate rate control of AF than from attempts to bring them back into sinus rhythm, thus further reducing the number of patients that truly benefit from chronic antiarrhythmic drug treatment.[9,10]

Although these data have changed our view on the pathophysiology, diagnosis, and treatment of arrhythmias dramatically, many of these studies do not apply to the emergency setting. In the emergency room or in the intensive care unit, drug treatment remains the gold standard for the treatment of cardiac arrhythmias. Arrhythmias are very common in emergency medicine, occurring in 12% to 20% of all patients in an intensive care unit,[11,12] and there is great need for good diagnostic and therapeutic algorithms to aid the emergency physician dealing with patients suffering from arrhythmias.

The first step towards effective acute treatment of arrhythmias in the emergency care setting is to arrive at a correct diagnosis. This is not always easy; often, the patient is hemodynamically unstable and there is not much time, an experienced electrophysiolgist is usually not close at hand, and advanced diagnostic equipment such as intracardiac ECG recordings may not be available. In their paper, Trappe *et al*.[13] nicely discuss the approach to the patient with various types of rhythm disturbances. They highlight the importance of obtaining a 12-lead ECG by any means possible. This has to be emphasized because a simple 12-lead ECG recording, together with some quick information on the patient's past medical history, may not only allow a correct arrhythmia diagnosis in most patients, it also has important implications for the patient's long-term prognosis after termination of the acute arrhythmia because the decision for further diagnostic or therapeutic interventions, such as an invasive electrophysiological study or an ICD implantation, may depend on that simple 12-lead ECG obtained during the index arrhythmia. It is still a frequent problem for electrophysiologists nowadays that patients are referred that have been treated only on the basis of a single-lead rhythm strip recording which may not even allow a clear differentiation between supraventricular and ventricular tachycardia. If there is no inducible arrhythmia during a later invasive electrophysiological study, it may be a very difficult to decide whether or not to implant a device.

In the same paper, the authors stress the importance of clarifying the underlying cardiac disease when an arrhythmic event occurs. In most instances, a life-threatening arrhythmia is only the symptom of a severe cardiac disease, e.g., ischemic heart disease, and treatment of precipitating factors for arrhythmic events (such as ischemia or electrolyte disturbance) should be considered even before antiarrhythmic drugs are given.

The correct use of antiarrhythmic drugs very often causes great confusion among physicians in training. Several antiarrhythmic drug classifications have been proposed[14,15] although the Vaughan-Williams classification remains the most widely used. In fact, the disappointing results with many antiarrhythmic drug trials had one positive consequence: the management of cardiac arrhythmias has become somewhat easier due to the limited number of drugs that have truly shown benefit. In this context, the second paper by Trappe *et al*.[16] provides a very interesting and simplified approach to the treatment of both tachy- and bradyarrhythmias. The proposed ‘5A’ concept focuses on the drugs adenosine, ajmaline, amiodarone, adrenaline and atropine that can be used to treat the vast majority of arrhythmias in the acute setting. Whereas adenosine, due to its short-term AV nodal blocking properties, has its major role in the termination of atrioventricular reciprocating tachycardias or in the unmasking of atrial tachycardias, ajmaline and amiodarone may be used for termination of ventricular tachycardia as well as acute stabilization in the presence of frequent VT recurrences. Interestingly, a prospective randomized comparison of amiodarone *vs* ajmaline or procainamide in this setting has never been published. In accordance with current guidelines,[17] amiodarone is preferred by most physicians due its high efficacy and the lack of negative inotropy and virtually no proarrhythmic effects. However, in the author's experience amiodarone takes a longer time to terminate an ongoing VT as compared to ajmaline in the emergency setting. Moreover, the potential side effects of the drug have to be taken into consideration even in the acute setting, as has been nicely pointed out by Trappe *et al*.

Apart from these antiarrhythmic drugs, the importance of two ‘old’ drugs, adrenaline and atropine, are also discussed by the authors. Whereas adrenaline has undoubted value in the setting of cardiac arrest due to either ventricular fibrillation, asystole, or electromechanical dissociation, atropine is still the drug of choice in sinus bradycardia or bradycardia due to advanced atrioventricular nodal block but not in the setting of a cardiac arrest.

Although the ‘5A’ concept is appealing, some important facts need to be stressed. Firstly, antiarrhythmic drugs known to be proarrhythmic, e.g., sodium channel blockers, may be safe and useful in a patient with unstable arrhythmias as long as he or she is under surveillance in an emergency care setting, although these compounds may not be prescribed for chronic treatment in patients with structural heart disease. This may apply, for example, to a patient who is still experiencing frequent VT recurrences despite being on adequate amiodarone dosage if he or she is not a candidate for VT ablation (see below). Secondly, an increasing number of patients are seen in the emergency room who already have an ICD implanted but who continue to suffer from frequent arrhythmia recurrences (arrhythmia ‘storms’) that lead to painful ICD discharges. In these patients, nearly every drug that suppresses frequent VT spells may be welcome in order to achieve a stable situation until a decision regarding long-term treatment can be made. In individual cases, patients with an implanted ICD may even need to remain on long-term treatment with class I drugs to prevent frequent VT recurrences. In this situation, potentially proarrhythmic drugs may be useful to reduce arrhythmia recurrences, with the ICD providing an electrical ‘backup’ in case the arrhythmia should indeed occur. Thirdly, in rare cases, catheter ablation may be a useful tool also in the emergency setting. Interestingly, the best success rates for radiofrequency catheter ablation of VT have been reported in incessant VT, a situation that usually mandates an emergency procedure.[18] The role that catheter ablation can play in an emergency care setting is highlighted by the case studies presented by Tebbenjohanns *et al*.[19] These cases indicate that catheter ablation nowadays may not only be a curative approach in the rare patient with aborted sudden cardiac death caused by an accessory pathway but may also dramatically improve symptoms in patients with incessant VT or arrhythmia ‘storm.’ Most fascinating, invasive electrophysiology may detect the occasional patient with idiopathic ventricular fibrillation in whom VT often originates at a site where Purkinje potentials can be recorded, and ablation at such sites may lead to abolishment of the frequent triggering beats initiating the ventricular fibrillation episodes.

Another setting in which there is still substantial uncertainty about the optimal approach to antiarrhythmic drug treatment is maternal or fetal arrhythmias during pregnancy. In their review, Trappe *et al*.[20] point out that in fact antiarrhythmic treatment during pregnancy follows the same general rules that apply for the general patient population with arrhythmias. There are three major aspects that deserve consideration during pregnancy: 1) potential teratogenic effects of specific antiarrhythmic agents need to be taken into consideration; 2) this requires a multidisciplinary approach, with the cardiologist, the gynecologist, and the neonatologist being involved in the decision making; and 3) a sound knowledge of the underlying cardiac disease (if any) and the type of arrhythmia to be treated (12-lead ECG!) is a prerequisite for an effective and safe treatment of the patient and the fetus.

In summary, although we have learned a lot about chronic antiarrhythmic drug treatment and non-phar macological alternatives in recent years, there is still some uncertainty about how and when antiarrhythmics should be used in the acute setting and which drug should be preferred for a given arrhythmia. The articles published in this issue of the Journal of Emergencies, Trauma, and Shock provides some practical guidance for the right approach to the diagnosis and treatment of most of the common arrhythmias encountered in the emergency room or the intensive care unit; the non-pharmacological alternatives are also covered. These reviews are likely to make life much easier for those not caring for arrhythmia patients everyday.
